# Diagnostic Yield of CE-EBUS in Mediastinal and Hilar Lymphadenopathy: A Preliminary Study †

**DOI:** 10.3390/jcm14082800

**Published:** 2025-04-18

**Authors:** Ilaria Suriano, Luca Frasca, Filippo Longo, Antonio Sarubbi, Giovanni Tacchi, Pierfilippo Crucitti

**Affiliations:** 1 Department of Thoracic Surgery, Fondazione Policlinico Universitario Campus Bio-Medico, Via Alvaro del Portillo, 200, 00128 Rome, Italy; filippo.longo@policlinicocampus.it (F.L.); antonio.sarubbi@alcampus.it (A.S.); giov.tacchi@outlook.it (G.T.); p.crucitti@policlinicocampus.it (P.C.); 2 PhD Course in Microbiology, Immunology, Infectious Diseases, and Transplants (MIMIT), University Tor Vergata, Viale Oxford, 81, 00133 Rome, Italy; 3 Master’s Degree Program in Medicine and Surgery, Campus Bio-Medico University, Via Alvaro del Portillo, 21, 00128 Rome, Italy

**Keywords:** EBUS, CE-EBUS, sulfur hexafluoride, mediastinal staging, lung cancer

## Abstract

**Background/Objectives**: Contrast-enhanced endobronchial ultrasound (CE-EBUS) is a minimally invasive technique that combines traditional endobronchial ultrasound (EBUS) with a contrast agent (sulfur hexafluoride), enhancing the visualization of blood flow in mediastinal and hilar lymph nodes. This study aimed to assess the use of CE-EBUS in patients with advanced neoplasms and hilar or mediastinal lymphadenopathy, particularly to improve diagnostic accuracy and expedite sample collection. **Methods**: A retrospective observational study was conducted from April 2021 to December 2023, involving 49 patients divided into two groups: EBUS (*n* = 26) and CE-EBUS (*n* = 23). Patients had advanced neoplasms with hilar and mediastinal lymphadenopathy, including bulky masses and nodal metastases with central necrosis. In the CE-EBUS group, 4.8 mL of sulfur hexafluoride was administered intravenously. Morphological, echogenic, and vascular characteristics, diagnostic accuracy, sample collection adequacy and molecular testing were compared between the groups. **Results**: The diagnostic accuracy in CE-EBUS was similar to EBUS (21 vs. 19 patients), with no significant difference (*p* = 0.100). However, for patients with bulky masses and necrosis, the molecular assessment rate was significantly higher in the CE-EBUS group (81.8%) compared to the EBUS group (33.3%) (*p* = 0.014). **Conclusions**: CE-EBUS-TBNA could improve the accuracy of molecular assessments in patients with bulky, necrotic lymphadenopathy and could help collect vital neoplastic tissue for molecular testing.

## 1. Introduction

Accurate staging of mediastinal lymph nodes is crucial to guide appropriate treatment strategies in patients with lung cancer. Conventional endobronchial ultrasound-guided transbronchial needle aspiration (EBUS-TBNA) is a cornerstone in the diagnosis and staging of mediastinal lymphadenopathy [1]. The implementation of updated innovative procedural and anesthetic protocols has been shown to enhance patient outcomes. Contrast-enhanced endobronchial ultrasound (CE-EBUS) is a minimally invasive technique that combines the traditional endobronchial ultrasound (EBUS) with the administration of a contrast agent (sulfur hexafluoride) that enhances the visualization of blood flow within mediastinal and hilar lymph nodes, potentially aiding in the identification of metastatic disease [2]. The use of this contrast has been widely investigated, particularly in procedures like endoscopic ultrasound (EUS) and transcutaneous B-mode ultrasound [3,4]. In fact, ultrasound contrast agents (UCAs) are used to improve characterization of microvascular and perfusion in various organs, helping differentiation of benign and malignant focal lesions and aiding in guiding biopsy [5]. Introduced over a decade ago, this imaging technique has been extensively studied, discussed, and incorporated into numerous clinical guidelines [6,7].

We evaluated the use of CE-EBUS in patients affected by advanced neoplasm with hilar and mediastinal lymphadenopathy to enhance the characterization of mediastinal lymph nodes and optimize biopsy target selection. The aim of this study is to propose the use of this technique in order to improve diagnostic accuracy and expedite sample collection.

## 2. Materials and Methods

We conducted a retrospective observational study from April 2021 to December 2023, collecting 49 patients affected by suspected or confirmed lung cancer involving mediastinal lymph nodes, hilar masses, and bulky (maximum diameter > 3 cm) lymphadenopathy, as well as nodal metastases exhibiting central necrosis. Patients with confirmed or suspected autoimmune or lymphoproliferative diseases were excluded from the study. All participants underwent a preoperative CT scan and 18F-FDG PET scan showing uptake in one or more mediastinal or hilar lymph nodes (SUV max > 2.5). Patients had lesions on CT scans considered central, peripheral, and hilar sized on their long axis [8,9]. Malignant features on CT scans, including absent hilum, necrosis, and irregular margins, were also assessed. Patients were divided into two groups: EBUS (*n* = 26) and CE-EBUS (*n* = 23). In the CE-EBUS group, 4.8 mL of sulfur hexafluoride was intravenously (IV) administered. According to our standard EBUS-TBNA protocol, each patient underwent a systematic exploration of the visible homolateral lymph node stations and of the contralateral hilum to exclude N3 (we also based it on the intra-procedural patient’s compliance). We compared morphological, echogenic, and vascular characteristics, as well as diagnostic accuracy, sample adequacy, and suitability for further molecular testing between the two groups. The study was conducted in accordance with the Declaration of Helsinki and with the approval of the Internal Review Board.

### 2.1. CE-EBUS Examination

Currently, there is no standardized protocol for CE-EBUS. To enhance the reliability of lymph node biopsies, we outline our proposed protocol for this procedure. Written informed consent was obtained from all patients prior to the procedure.

#### 2.1.1. Anesthesia

Topical anesthesia was nebulized to the pharynx and upper airways using 2% lidocaine into the nose and mouth. Thereafter, conscious sedation was performed 5 min before the endoscopic procedure with the infusion of IV remifentanil (1 ng/mL increased to 6 ng/mL) to reach a satisfying level of sedation. Continuous adjustments of anesthesia depth were necessary to ensure patient safety and operator’s comfort throughout the procedure.

#### 2.1.2. Procedure

The procedure involved the IV injection of a 4.8 mL sulfur hexafluoride bolus as an UCA via a peripheral vein cannula. Contrast was injected for each lymph node or mass on which the biopsy was performed. After 16 and 17 s post-contrast injection, significant enhancement of the vascular pattern is evident, and the decrease in enhancement is 32 s post-injection [10]. Specimens were collected using a 22-gauge aspirating-cutting needle (SonoTip TopGain^®^ EBUS with stainless steel L-GUB-45-18-022, Medi-Globe GmbH Rohrdorf/Achenmühle, Germany), which was maneuvered back and forth through the lymph node [11]. At least three passes were made for each lymph node [11]. This was guided by a convex endobronchial ultrasound (EBUS) (EB-530US Fujifilm Echobronchoscope^®,^ Lexington, MA, USA) equipped with an endoscopic ultrasound scanner at a frequency of 10 MHz. Real-time imaging allowed for detailed analysis of lymph node features, including morphology, echogenicity, and tissue characteristics. This facilitated the assessment of micro vessel architecture and neo-angiogenesis, as well as the detection of potential necrosis. Finally, adequate specimens were collected for histological analysis. Patients were monitored for three hours after the end of the procedure.

#### 2.1.3. Pathology

Specimens collected through the procedure were subjected to genetic testing using the following assays:

- Myriapod NGS Cancer Panel DNA Assay Kit (Diatech Pharmacogenetics, CE-IVD Kit, Jesi, Italy), which requires a minimum of 20–25 ng of DNA per sample for a successful analysis.
- VENTANA PD-L1 (SP263) Assay (CE-IVD).

A result was considered successful only if both assays were completed for the collected specimens; therefore, a sufficient quantity of material needed to be obtained.

### 2.2. Statistical Analysis

Categorical variables were presented as number and percentage (*n*, %) while continuous variables were presented as median and interquartile range (IQR). Diagnostic accuracy was estimated as the percentage of biopsies allowing a histology diagnosis in both groups. Differences between the CE-EBUS and EBUS groups were assessed using Fisher’s exact test or the chi-square test. We also performed a sub-analysis for patients presenting bulky masses and lesions showing areas of necrosis and compared the results of both techniques in terms of diagnostic accuracy and molecular assessment rate. We considered significant *p*-values < 0.05. All statistical analyses were performed with Jamovi software version 2.3.28.0.

## 3. Results

The final study population consisted of 49 patients. For all patients, three passes were performed per lymph node, except for two patients in the EBUS group and one in the CE-EBUS group, who required four passes due to macroscopic sample inadequacy. In the comparison between the EBUS group and the CE-EBUS group, the median age of patients was 70 ± 9.2 (IQR) in the EBUS group and 69.8 ± 8.4 (IQR) in the CE-EBUS group. Regarding histology, adenocarcinoma was identified in 50% of patients in the EBUS group compared to 34.8% in the CE-EBUS group, while squamous cell carcinoma was observed in 7.7% and 8.7% of patients, respectively. Clinical staging covered stages IIB, IIIA, IIIB, IIIC, IVA, and IVB. Patient characteristics are summarized in Table 1.

Morphological characteristics are shown in Table 2.

In the CE-EBUS group, 16 patients had bulky lesions, compared to 16 patients in the EBUS group (69.6% vs. 61.5%). Coagulation necrosis sign was present in 17 patients in the CE-EBUS group and in 21 patients in the EBUS group (73.9% vs. 80.8%), while indistinct margins were present in 14 patients in the CE-EBUS group and 19 patients in the EBUS group (60.9% vs. 73.1%). Echogenicity results were heterogeneous in 20 patients undergoing CE-EBUS and in 25 patients undergoing EBUS (87% vs. 96.2%).

**Figure 1:**
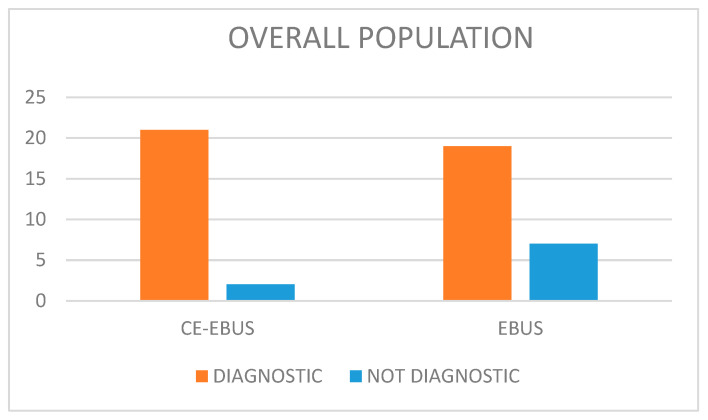
Distribution of diagnostic and nondiagnostic biopsies in the CE-EBUS and EBUS groups.

Diagnostic accuracy in the overall population in the EBUS and CE-EBUS groups is shown in Table 3 and Figure 1.

For patients showing bulky masses and necrosis presence, diagnostic biopsies were successful in 10 patients in the CE-EBUS group versus 14 patients in the EBUS group, showing no significance in terms of diagnostic accuracy between the two groups (*p* = 0.819). But in this kind of patient, the CE-EBUS group demonstrated a higher molecular assessment rate (PDL-1, EGFR, KRAF, BRAF) in specimens collected with a success of 81.8% compared to 33.3% in the EBUS group (*p* = 0.014) (Table 4).

## 4. Discussion

The role of EBUS in the staging of lung cancer has become increasingly significant, and it remains a technique with a high diagnostic accuracy [12]. There is a growing demand for molecular investigations to support the use of appropriate oncological therapies in this field [13]. In fact, EBUS is used as an adjunct to guide the delivery of treatments used in the management of primary lung malignancy and as an adjunct in palliative treatments like therapeutic interventional bronchoscopy [14,15].

Effective EBUS-TBNA practice depends on both precise technique and well-defined protocols to ensure optimal sample quality for diagnosis and further analysis. The American College of Chest Physicians reviewed the literature on this procedure, highlighting the critical role of adequate tissue collection in evaluating suspected malignancies, including lung cancer [11]. In order to satisfy this request, many centers incorporate techniques such as cryobiopsy or elastography to enhance EBUS success and obtain a greater quantity of viable tissue from lymph node sampling [16,17,18]. Additionally, in patients with suspected malignant disease undergoing EBUS-TBNA, the use of rapid on-site evaluation (ROSE) has found more space to assess the adequacy of the biopsy sample [11,19]. However, these procedures entail high costs, and not all centers have access to these resources. In the ultrasound (US) evaluation of large neoplastic masses of various organs, a sulfur-based contrast agent has been used for years (CEUS) [19]. The use of this contrast makes it easier to identify viable areas to be biopsied by distinguishing them from necrotic areas [20]. With EBUS being an ultrasound-based procedure, we used the same contrast to optimize sampling of the hilar and mediastinal lymph nodes (CE-EBUS) [16].

In the literature, the feasibility and diagnostic potential of CE-EBUS-TBNA for mediastinal lymph node study has received limited attention. CEUS is commonly used to study microcirculation in organs such as the pancreas, liver, and breast [21]. Despite evidence suggesting that ultrasound contrast agents can improve the accuracy of percutaneous needle biopsies [22], the use of CE-EBUS remains underexplored. Dietrich et al. examined its role in assessing hilar or mediastinal lymph nodes [10]. Similarly, some authors have evaluated the use of sulfur-based contrast in EUS procedures for studying the mediastinum [23,24]. Cui et al. have investigated the utility of CEUS combined with EUS for differential diagnosis and reported that the identification of hypoenhanced areas in malignant lymph nodes may be helpful to guide biopsy [6]. In fact, this technique can provide detailed images of microvessel architecture and neo-angiogenesis, thus improving the detection of viable tissues and avoiding necrotic areas during biopsy [7]. Indeed, in our opinion, CE-EBUS may significantly improve the characterization of lymphadenopathies by producing higher-quality images. The use of Doppler ultrasound (US) and elastosonography can enhance the visualization of a lesion’s vascular pattern. In benign reactive lymph nodes, the vascular pattern and morphology remain preserved. In contrast, malignant lymph nodes are characterized by neovascularization, evident when vessels penetrate the capsule of the node away from the hilum, a distinguishing feature of metastatic lymph nodes. The visualization of vascular anatomy can be further improved with the use of UCA during color Doppler US. The addition of contrast enhances the intensity of Doppler signals (color Doppler and power Doppler), enabling the detection of slow, low-volume blood flow, e.g., tumor vessel [25].

**Figure 2:**
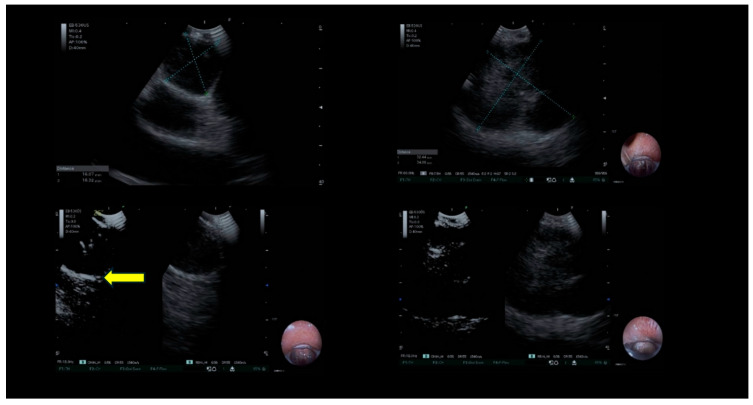
Contrast-enhanced EBUS-TBNA in mediastinal lymphadenopathy. Malignant bulky mediastinal lymph node. After 16–17 s post contrast injection, significant enhancement of the vascular pattern is evident (yellow arrow). The biopsy was performed avoiding non-enhanced areas indicative of necrosis. The left image shows a strip of hyperechoic tissue, corresponding to the vital tissue area where the biopsy was performed. In contrast, the B-mode image on the right does not clearly reveal this area.

On CE-EBUS, malignant infiltration was characterized by centripetal, inhomogeneous enhancement, alterations in the vascular architecture of microvessels, and the presence of avascular areas (Figure 2).

Indeed, obtaining a high-quality sample is essential given the increasing demand for detailed molecular testing. This is crucial not only for accurate staging, which significantly influences prognosis, but also for conducting a comprehensive analysis [13]. Comparing the two groups (EBUS and CE-EBUS), our data did not show a statistically significant difference in terms of diagnostic accuracy. Actually, in the two groups, the percentage of patients who were diagnosed did not differ either in terms of diagnosis or the possibility of performing molecular investigations. On the other hand, it is noteworthy that when examining patients with bulky adenopathies or extensive areas of necrosis, the two groups exhibit significant differences. In particular, CE-EBUS showed a higher success rate in obtaining sufficient viable tissue for both histological typing and molecular testing. This result is particularly interesting, as it shows how this contrast could be a good tool in cases where large areas of necrosis make it challenging to obtain a viable tissue sample. Consequently, this approach could reduce the need for repeat procedures. Despite the encouraging results, this study has significant limitations. First, it is a single-center retrospective study. Furthermore, the small cohort size limits the generalizability of our results. Considering these factors, it cannot be asserted that CE-EBUS could replace established procedures such as ROSE or cryobiopsy [26]. In conclusion, in view of the cheaper nature, the non-toxicity of the contrast medium, and the relatively quick learning curve, we consider it worthwhile to suggest further studies on this technique. In our opinion, it could be a viable option, especially in endoscopy centers where advanced pre-diagnostic tools are not available.

## 5. Conclusions

CE-EBUS-TBNA could improve the accuracy of molecular assessment, moreover, in patients presenting with extensively necrotic hilar and mediastinal lymph nodes. In addition, improved real-time ultrasound image quality from sulfur-based contrast has proven feasible and clinically safe, highlighting the need for further research to fully explore its diagnostic potential.

## Figures and Tables

**Table 1:** Patient’s characteristics.

Patient Characteristics	CE-EBUS, *n* (%) *n* = 23	EBUS, *n* (%) *n* = 26
Sex		
Male	11 (47.8)	16 (61.5)
Female	12 (52.2)	10 (38.5)
Age (median ± IQR)	69.8 ± 8.4	70 ± 9.2
Smoker		
Unknown	4 (17.4)	4 (15.4)
Current	7 (30.4)	7 (26.9)
Ceased	11 (47.8)	12 (46.1)
Never	1 (4.4)	3 (11.6)
Histology		
Adenocarcinoma	8 (34.8)	13 (50.0)
Squamous cell carcinoma	2 (8.7)	2 (7.7)
Large cell carcinoma	3 (13.0)	1 (3.8)
Small cell carcinoma	4 (17.4)	2 (7.7)
Large-cell neuroendocrine carcinoma	3 (13.0)	0
Other	1 (4.4)	1 (3.8)
Nondiagnostic	2 (8.7)	7 (26.9)
Tumor stage (*n*)		
Stage IIB	1 (4.4)	2 (7.7)
Stage IIIA	3 (13.0)	3 (11.6)
Stage IIIB	3 (13.0)	3 (11.6)
Stage IIIC	4 (17.4)	5 (19.2)
Stage IVA	5 (21.8)	4 (15.4)
Stage IVB	7 (30.4)	9 (34.5)
Tumor location on CT		
Central	3 (13.0)	5 (19.2)
Peripheral	15 (65.0)	17 (65.4)
Hilar	5 (22.0)	4 (15.4)
Tumor size on CT on long axis (cm)		
<3	3 (13.0)	4 (15.4)
3–5	7 (30.4)	11 (42.3)
5–7	11 (47.8)	10 (38.5)
>7	2 (8.7)	1 (3.8)
PET SUV max (*n*)		
2.5–3.9	3 (13.0)	3 (11.6)
4–7.9	11 (47.8)	14 (53.8)
>8	9 (39.2)	9 (34.6)
No uptake	0	0

IQR: interquartile range, CT: computed tomography, PET: positron emission tomography, SUV: standardized uptake value.

**Table 2:** Morphological characteristics of 49 patients divided into CE-EBUS and EBUS groups.

	CE-EBUS *n* (%) *n* = 23	EBUS *n* (%) *n* = 26	*p* Value
Size > 30 mm	16 (69.6)	16 (61.5)	0.55
Size < 30 mm	7 (30.4)	10 (38.4)	
Shape: round	1 (4.4)	3 (11.5)	0.81
Shape oval	22 (95.6)	23 (88.5)	
Margin: distinct	9 (39.1)	7 (26.9)	0.36
Margin: indistinct	14 (60.9)	19 (73.1)	
Central hilar structure: presence	4 (17.4)	4 (15.4)	0.77
Central hilar structure: absence	19 (82.6)	22 (84.6)	
Echogenicity: heterogenous	20 (87)	25 (96.2)	0.24
Echogenicity: homogeneous	3 (13)	1 (3.8)	
Coagulation necrosis sign: presence	17 (73.9)	21 (80.8)	0.56
Coagulation necrosis sign: absence	6 (26.1)	5 (19.2)	

**Table 3:** Diagnostic yields of EBUS-TBNA and CE-EBUS-TBNA on diagnostic accuracy in the overall population.

	CE-EBUS *n* (%)	EBUS *n* (%)	*p*-Value
Diagnostic biopsy	21 (91.3)	19 (73.1)	0.100
Nondiagnostic biopsy	2 (8.7)	7 (26.9)	

**Table 4:** Diagnostic yields and molecular assessment in bulky masses and necrosis presence population.

	CE-EBUS *n* (%)	EBUS *n* (%)	*p*-Value
Diagnostic biopsy	10 (90.9)	14 (93.3)	0.819
Nondiagnostic biopsy	1 (9.1)	1 (6.7)	
Molecular assessment (Yes)	9 (81.8)	5 (33.3)	0.014
Molecular assessment (No)	2 (18.2)	10 (66.7)	

## Data Availability

The datasets generated and/or analyzed during the current study are available from the corresponding author on reasonable request.
